# Value of 3D preoperative planning for primary total hip arthroplasty based on artificial intelligence technology

**DOI:** 10.1186/s13018-021-02294-9

**Published:** 2021-02-24

**Authors:** Jiabang Huo, Guangxin Huang, Dong Han, Xinjie Wang, Yufan Bu, Ya Chen, Daozhang Cai, Chang Zhao

**Affiliations:** 1 Department of Orthopedics, Orthopedic Hospital of Guangdong Province, The Third Affiliated Hospital of Southern Medical University, No. 183, Zhongshan Rd West, Guangzhou, 510630 China; 2 Department of Quality Management and Evaluation, Orthopedic Hospital of Guangdong Province, The Third Affiliated Hospital of Southern Medical University, Guangzhou, Guangdong China

**Keywords:** AI, Artificial intelligence, Preoperative planning, Total hip arthroplasty, THA

## Abstract

**Background:**

Accurate preoperative planning is an important step for accurate reconstruction in total hip arthroplasty (THA). Presently, preoperative planning is completed using either a two-dimensional (2D) template or three-dimensional (3D) mimics software. With the development of artificial intelligence (AI) technology, AI HIP, a planning software based on AI technology, can quickly and automatically identify acetabular and femur morphology, and automatically match the optimal prosthesis size. However, the accuracy and feasibility of its clinical application still needs to be further verified. The purposes of this study were to investigate the accuracy and time efficiency of AI HIP in preoperative planning for primary THA, compared with 3D mimics software and 2D digital template, and further analyze the factors that influence the accuracy of AI HIP.

**Methods:**

A prospective study was conducted on 53 consecutive patients (59 hips) undergoing primary THA with cementless prostheses in our department. All preoperative planning was completed using AI HIP as well as 3D mimics and 2D digital template. The predicted component size and the actual implantation results were compared to determine the accuracy. The templating time was compared to determine the efficiency. Furthermore, the potential factors influencing the accuracy of AI HIP were analyzed including sex, body mass index (BMI), and hip dysplasia.

**Results:**

The accuracy of predicting the size of acetabular cup and femoral stem was 74.58% and 71.19%, respectively, for AI HIP; 71.19% (*P* = 0.743) and 76.27% (*P* = 0.468), respectively, for 3D mimics; and 40.68% (*P* < 0.001) and 49.15% (*P* = 0.021), respectively, for 2D digital templating. The templating time using AI HIP was 3.91 ± 0.64 min, which was equivalent to 2D digital templates (2.96 ± 0.48 min, *P* < 0.001), but shorter than 3D mimics (32.07 ± 2.41 min, *P* < 0.001). Acetabular dysplasia (*P* = 0.021), rather than sex and BMI, was an influential factor in the accuracy of AI HIP templating. Compared to patients with developmental dysplasia of the hip (DDH), the accuracy of acetabular cup in the non-DDH group was better (*P* = 0.021), but the difference in the accuracy of the femoral stem between the two groups was statistically insignificant (*P* = 0.062).

**Conclusion:**

AI HIP showed excellent reliability for component size in THA. Acetabular dysplasia may affect the accuracy of AI HIP templating.

## Background

Total hip arthroplasty (THA) is a successful orthopedic surgery that can reduce pain and improve joint function. However, when the prosthesis is improperly selected, it will lead to a series of intraoperative and postoperative complications, such as leg length discrepancy [1], joint dislocation [2], periprosthetic fracture [3], aspetic loosening [4, 5], and stress-shielding [6, 7]. This will lead to an increase in the revision rate, resulting in patient dissatisfaction and lower clinical score.

Therefore, a successful THA requires the selection of the most suitable prosthesis size and restoration of joint biomechanics. Accurate preoperative planning can help obtain information about reconstruction results in advance [8]. Although navigation and robots can provide relatively accurate preoperative planning, they are time-consuming and costly, with high error rates of the manual system, and long learning curve involved [9]. Therefore, preoperative planning with navigation or robots cannot be an ideal conventional application technology at present. At present, 2D templates are mostly used in clinical practice, but their accuracy is low due to the influence of X-ray magnification [10] and projection position [11]. Petretta et al. [12] compared the preoperative planning of 260 patients with the film template method and digital template; the complete accuracy rate of both methods was only 28%. Holzer et al. [13] conducted a retrospective study on 632 patients using a digital template, and the complete accuracy rate of predicting acetabular cup and femoral stem was 37% and 42%, respectively. While Efe [14] for 169 patients using a digital template was 33.7% and 36%, respectively. A few hospitals use 3D planning software for preoperative planning of THA. Although the accuracy is relatively high, the acetabular rotation center, femoral osteotomy position, and femoral stem implantation depth, which require constant manual adjustment and cumbersome operation, could not be located. Zeng et al. [15] used 3D mimics to make preoperative planning for 20 patients with Crowe IV hip dysplasia, and the complete accuracy of acetabular cup was 70% and 100% within one size. Sariali et al. [16] used 3D software for preoperative planning of 30 patients receiving THA, and the accuracy of predicting one size of the femoral stem and acetabular cup was 100% and 96%, respectively. Inoue et al. [17] used 3D planning software Zed hip to plan 65 hips of 57 patients. The complete accuracies of acetabular cup and femoral stem were 92% and 65%, respectively, and the accuracy rate of one acetabular cup and femoral stem was 100% and 98%, respectively.

The 2D and 3D planning methods mentioned are subjective or time-consuming. Therefore, to achieve rapid planning and avoid planning deviation caused by personal experience is the key problem to be solved in preoperative planning. With the development of artificial intelligence (AI) technology, AI HIP, a planning software based on AI technology, can quickly and automatically identify acetabular and femur morphology, and automatically match the optimal prosthesis size. However, the accuracy and feasibility of its clinical application still needs to be further verified. As far as we know, the accuracy of AI HIP has not been studied.

Therefore, we carried out a prospective study to evaluate (1) the accuracy and time efficiency of AI HIP and compare with the 3D mimics and the 2D digital template and (2) the factors that influence the accuracy of AI HIP: Sex, BMI, and hip dysplasia.

## Materials and methods

This was a prospective clinical study to assess the accuracy of AI HIP in primary THA and to further analyze the factors influencing its accuracy. The research protocol was approved by the institutional review board and all participants gave their written informed consent.

From October 2019 to July 2020, preoperative planning was performed for every patient who underwent primary THA with cementless prostheses. All included patients underwent preoperative CT scans and routine X-ray examination. CT scan, ranging from the upper edge of pelvis to at least 10 cm distal to the lesser trochanter, was carried out with a Toshiba Aquilion 64 CT scanner (voltage: 120 kVp, electric current: intelligent mA control, according to the patient fat and thin draw mA curve, CT matrix: 512 × 512 matrix, slice thickness: 1 mm). And the CT images were stored in DICOM format.

On the eve of the surgery, each patient was planned using AI HIP, 3D mimics, and 2D digital template, which were divided into three groups: AI, 3D, and 2D groups. The results of each group were recorded, including acetabular cup size, femoral stem size, and templating time. The templating time is the period from the beginning of importing data to the end of planning. All THAs were performed by the same senior arthroplasty surgeon, using the same posterolateral approach. The actual size of the prosthesis implanted during the operation was obtained from surgical records. The preoperative planning results of each group were compared with the actual intraoperative implant sizes to determine their accuracy. In this study, all preoperative planning was performed by the author. To identify planning reliability, all plannings were checked by two experienced surgeons. If there was a disagreement, the planning was repeated with all the three surveyors agreed upon.

To ensure that the surgeon was not affected by the preoperative planning results during the surgery and to maintain the consistency between the planning and the surgery, the surgeon was blind to the planning results.

### Preoperative planning

#### AI group preoperative planning

##### The working principle of AI HIP

**Fig. 1:**
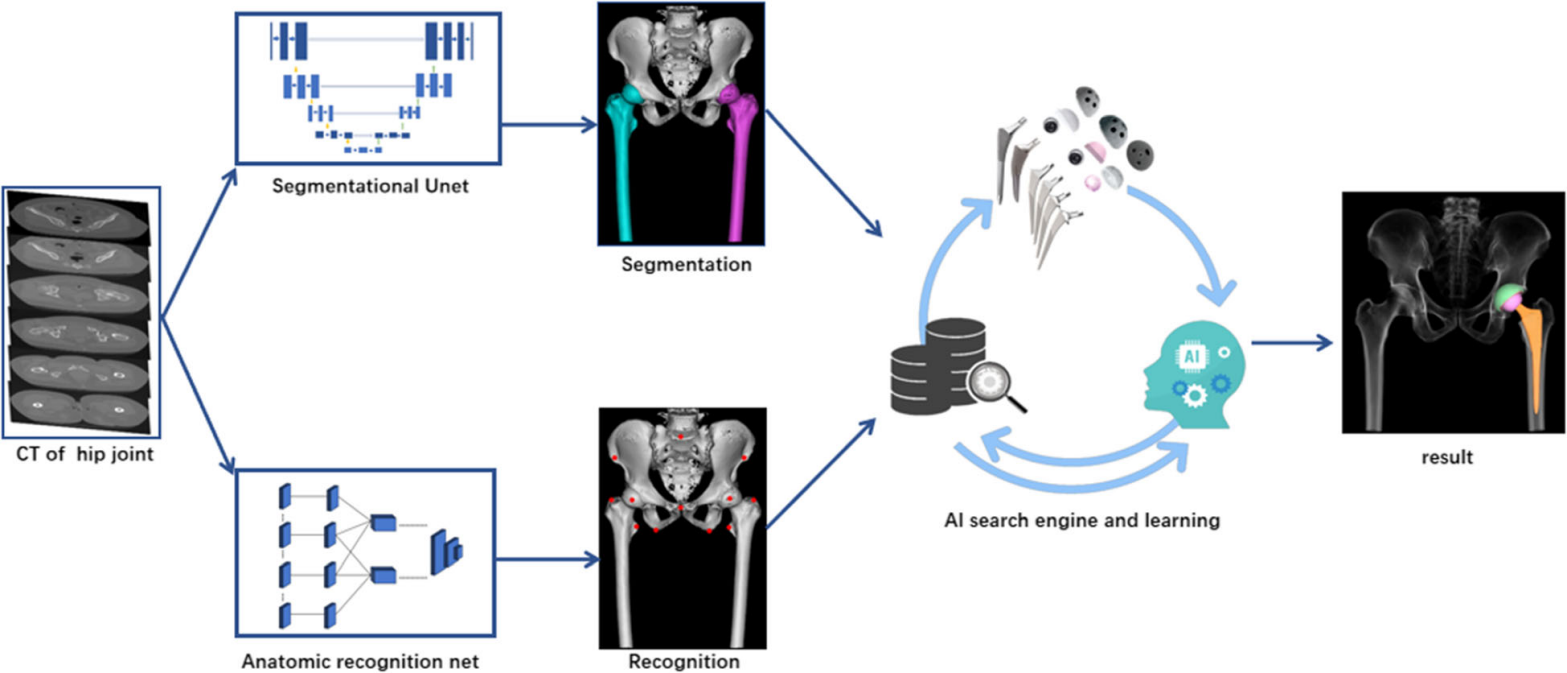
The flow chart diagram showed the working principle of AI HIP

The raw CT data were input into the segmentation model and 3D recognition model, both of which are deep learning models based on neural networks. The segmentation module separates the different parts of the bone and uses a multiple 3D segmentation neural network (using Unet as baseline) to ensure segmentation accuracy, dividing the bone into different areas such as the pelvic and femur areas. The anatomical point recognition module uses the point recognition neural network of face recognition and pattern recognition technology to accurately calculate the coordinates of corresponding points, locate the key points on the skeleton, and output the coordinates of the points, such as the lesser trochanter, greater trochanter, tear drop, and anterior superior iliac spine. Then, AI HIP matches the optimal prosthesis through an automatic search engine based on big data and reinforcement learning, and finally, the perfect intelligent results are planned (Fig. 1).

##### Planning process

**Fig. 2:**
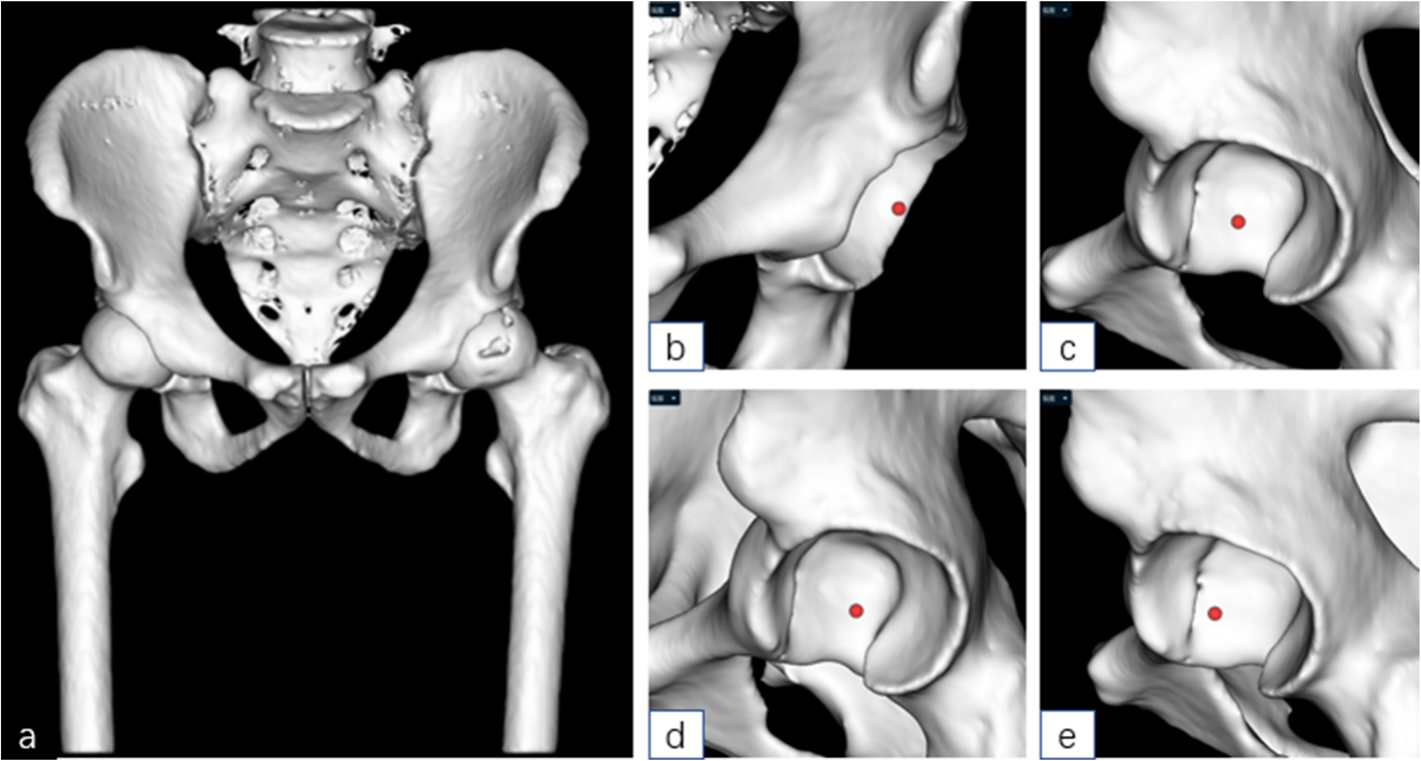
Schematic diagram of 3D modelings of AI HIP

**Fig. 3:**
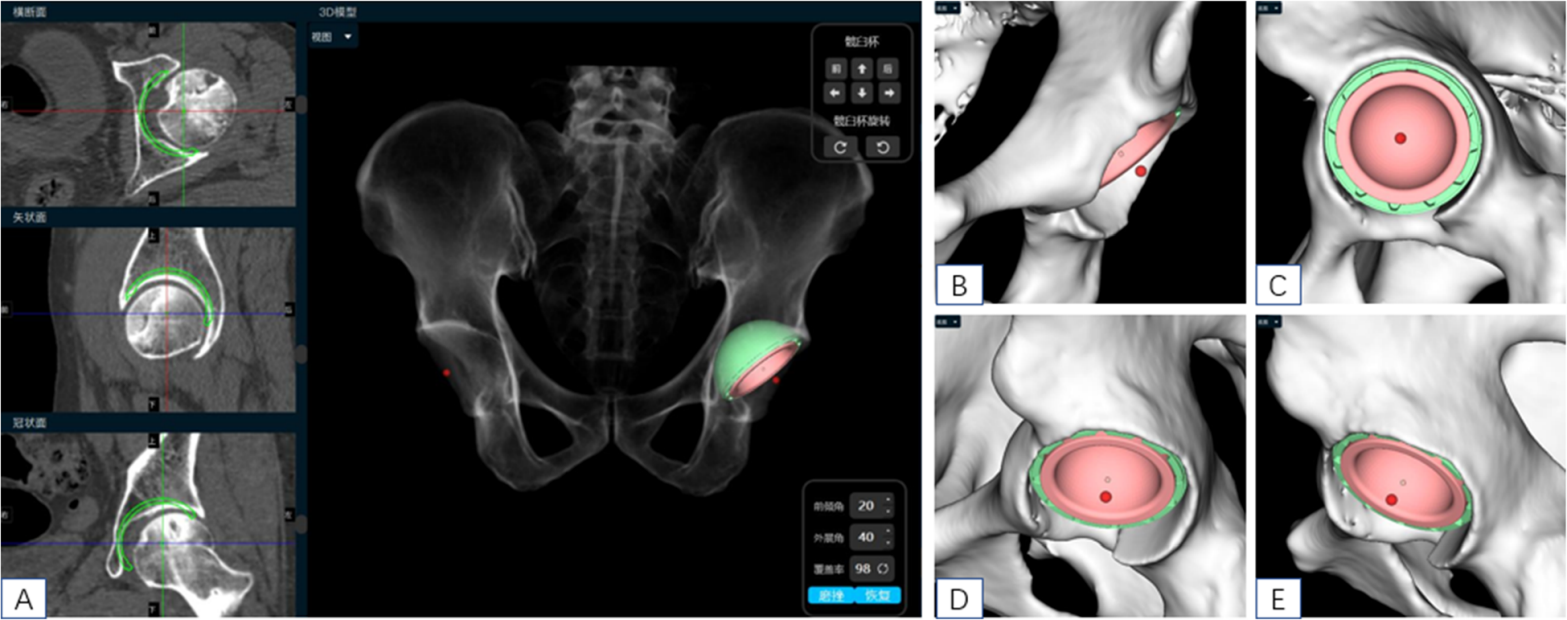
Schematic diagram of simulation of cup placement by AI HIP

**Fig. 4:**
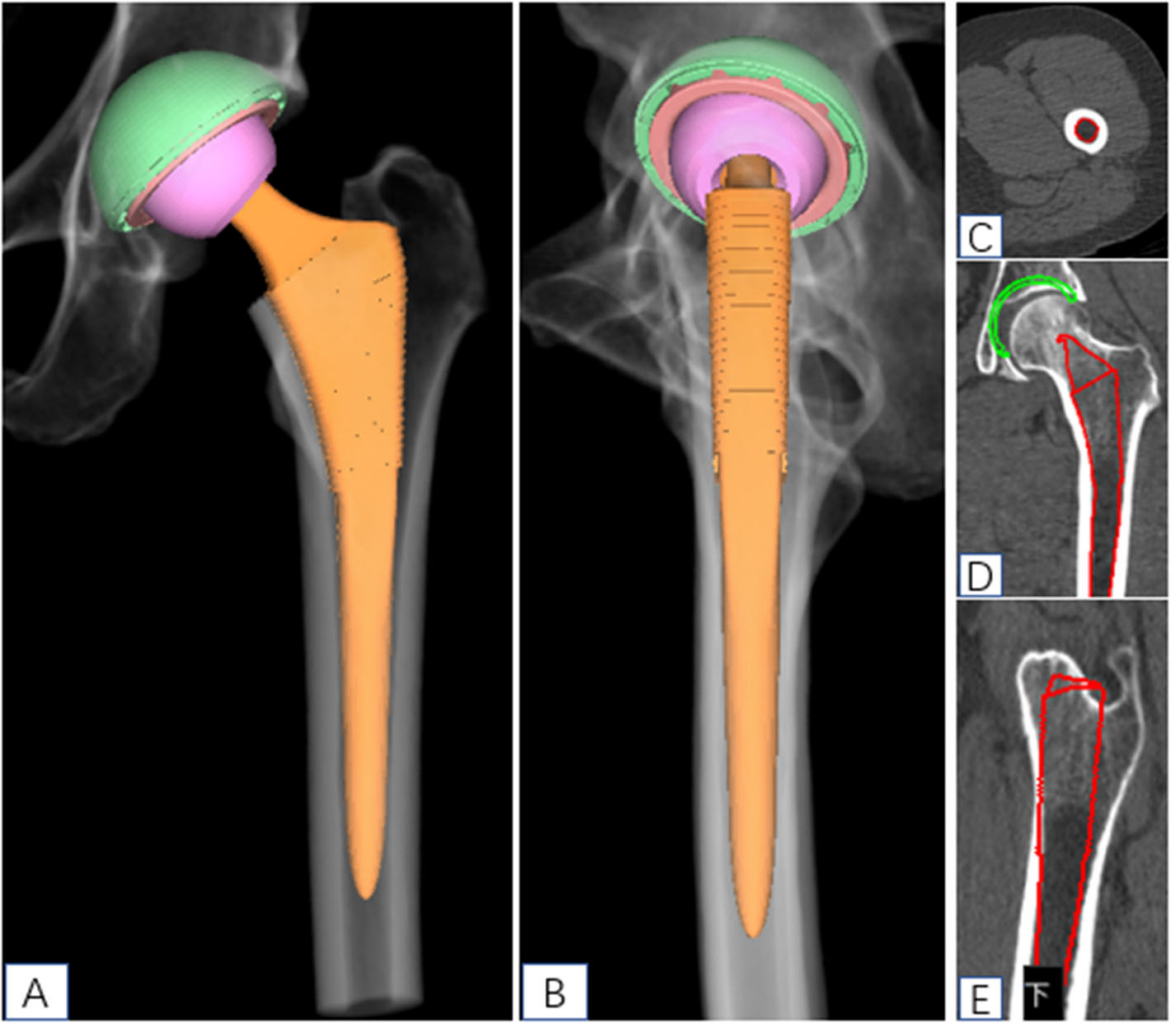
Schematic diagram of simulation of femoral stem placement by AI HIP

**Fig. 5:**
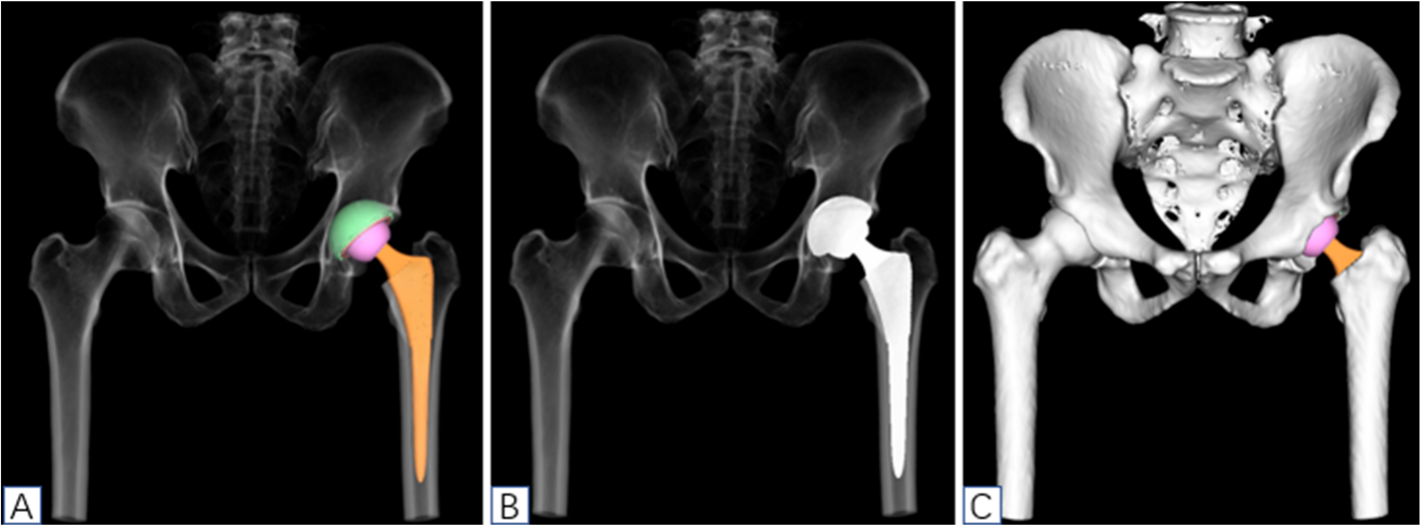
Images showed AI HIP multi-view observation planning

The DICOM format of the hip CT data was converted to “.cmg” format, the “.cmg” format image data of the patient was imported into AI HIP software (Changmugu), which automatically segmented the bones through AI technology and established 3D models of the pelvis and femur. Through amplification or rotation, the 3D images could be visualized and the severity of the lesions could be grasped (Fig. 2). Through neural network, automatic correction of the pelvis and simulating pelvic anteroposterior X-ray, leg length discrepancy, and femoral offset were calculated automatically. According to the modeling and calculation before, according to the automatic recognition of acetabular anteroposterior diameter and upper and lower diameter, the optimal acetabular cup size was selected from the prosthesis database and automatically matched to the acetabular socket model. The acetabular cup is automatically placed with an anteversion angle of 20° and an abduction angle of 40° so that the acetabular coverage rate can be calculated automatically. The coverage rate can change with real-time changes in the abduction angle and anteversion angle. The appearance and size of the prosthesis in the database are exactly the same as the prosthesis used in the operation, and there is no prosthesis deviation. If you are not satisfied with the automatic placement of AI, you can fine-tune it. Based on the automatic recognition of bone markers such as the epiphysis of the femoral shaft and the inner and outer cortex of the femoral shaft, the software selected the optimal femoral stem size from the prosthesis database, matched it automatically to the 3D model, and the femoral head size was selected according to the size of the acetabular cup (Fig. 3). After the prosthesis was placed, the leg length difference and offset under ideal conditions could be calculated automatically. At this time, femoral neck osteotomy could be performed. Finally, after the acetabular cup, femoral stem, and femoral head were placed, the software automatically calculated the length difference of the lower limbs and the height of the osteotomy (distance from the osteotomy plane to the tip of the lesser trochanter) and simulated the effect of pelvic anteroposterior radiography after operation (Figs. 4 and 5).

#### 3D group preoperative planning

##### Fabrication of 3D prosthesis

In order to make the shape and size of the 3D prosthesis consistent with the prosthesis prototype used in surgery, we used a 3D scanner (Epson E-018) to scan the PINACLE acetabular cup, SUMMIT, and CORAIL stem of various sizes. The original size model was established by reverse modeling with Geomagic Studio 2012 (Raindrop) and saved in STL format.

##### Planning process

**Fig. 6:**
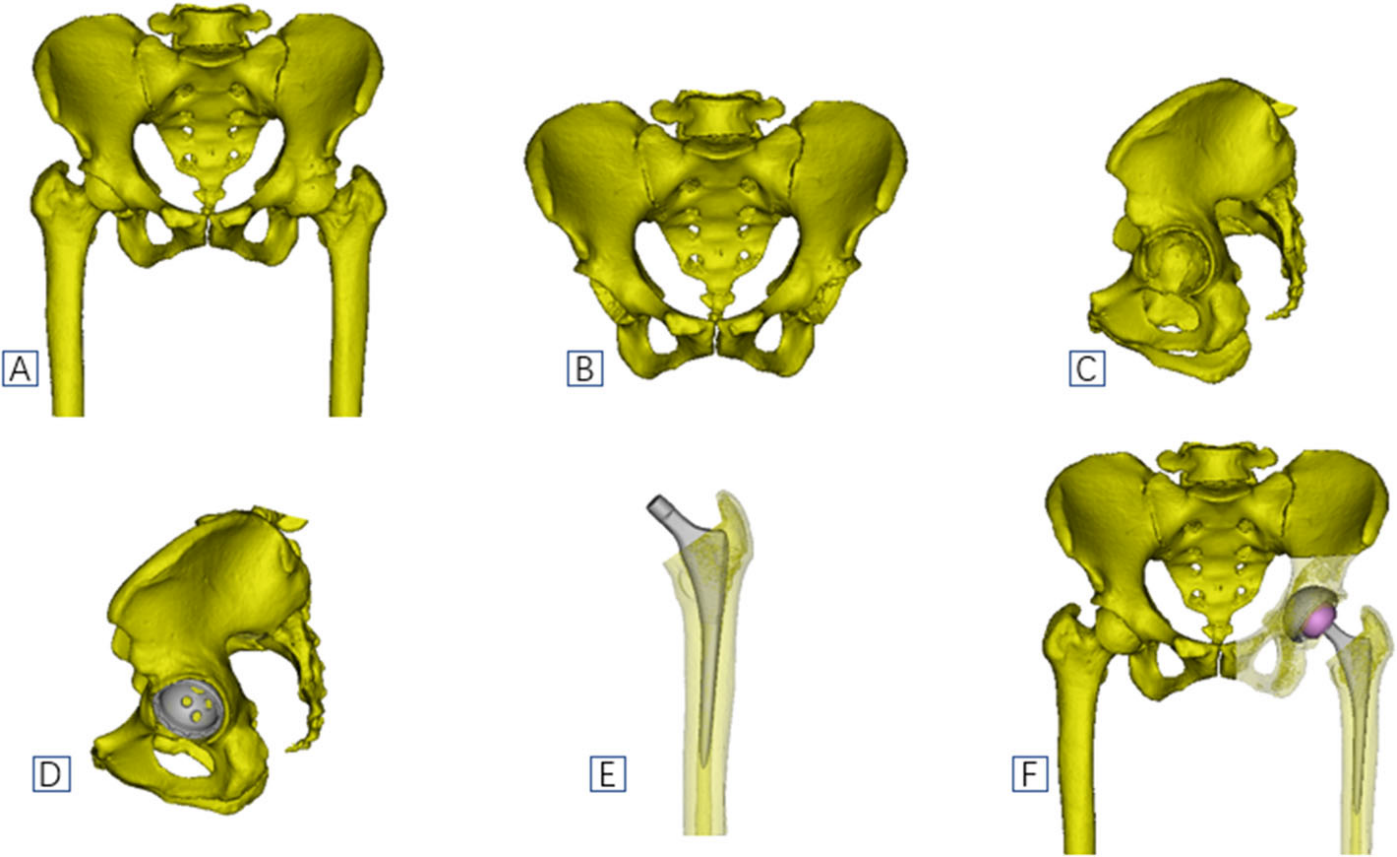
Schematic diagram of cup and stem planning by 3D mimics

The patient CT data in DICOM format were imported into mimics19 software (materialize). The original mask of the bone was selected by a threshold segmentation function. The pelvis and femur were segmented by region growing, editing mask function, and Boolean operation. The 3D model was then reconstructed. First, simulate the acetabular cup implantation, measure the anterior and posterior diameter length of the acetabulum, select the PINNACLE 3D cup with similar anterior and posterior diameter lengths to import into mimics in STL format, and make the 3D cup adhere to the anterior and posterior wall of the acetabulum on the transverse section, the coronal, sagittal, and select the optimal size of the acetabular cup after repeated adjustments on the coronal, sagittal, and 3D pelvic models [18, 19]. The femoral stem was then planned, the STL format of the SUMMIT or CORAIL femoral stem was imported, and the best prosthesis fitted closely with the epiphyseal cortex of the femoral shaft [17, 20]. The appropriate size of the femoral head was selected, based on the size of the acetabular cup (Fig. 6).

#### 2D group preoperative planning

**Fig. 7:**
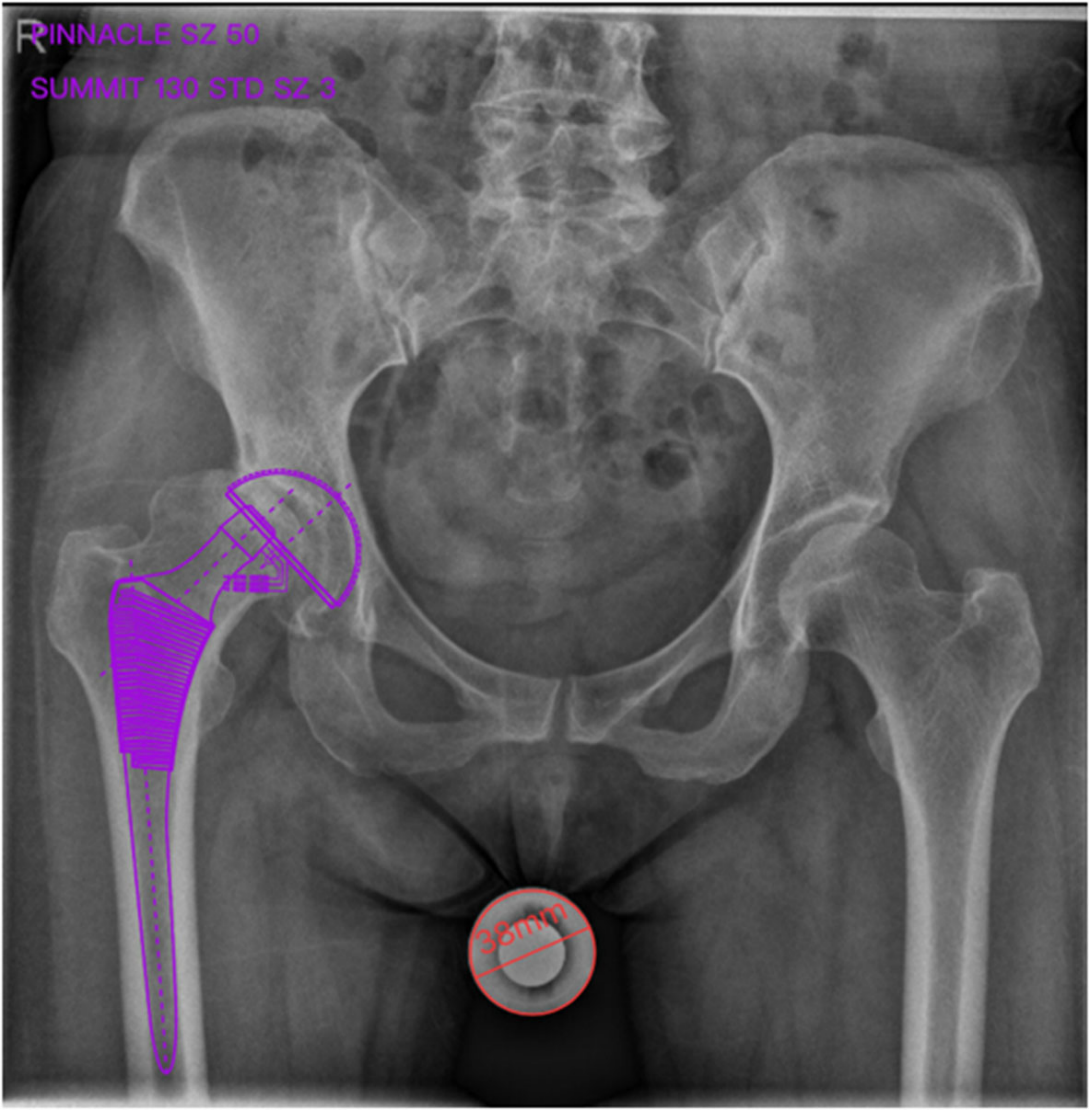
Schematic diagram of cup and stem planning by 2D digital templating

2D digital templating was based on the AP pelvic X-ray, which was performed in a standard manner with a 38-mm magnification marker ball in the perineum area at the level of the greater trochanter. The patient’s standard pelvic anteroposterior X-ray was imported into the 2D digital template (Smart joint, Depuy Synthes), and the diameter of the marker ball was set to 38 mm. The digital template prosthesis was superimposed on the AP pelvic radiograph, and the optimal size of the prosthesis was selected according to the matching situation between the acetabular cup and the acetabulum, femoral stem, and femoral medullary cavity [21] (Fig. 7).

Finally, the accuracy and time efficiency of the AI and 3D groups, AI group, and 2D group were compared, and the factors influencing the accuracy of AI HIP were further analyzed, mainly from three aspects: (1) sex and (2) BMI (according to the CHINA standard: normal: 18.5 ~ 23.9, pre-overweight/obese: 24–27.9, obesity: ≥ 28) and hip dysplasia (developmental dysplasia of the hip (DDH) group and non-developmental dysplasia of the hip (non-DDH) group).

### Statistical analysis

Quantitative data are described as mean ± standard deviation, while categorical variables are described as counts (percentage). The prediction was considered accurate if it was consistent with the size used intraoperatively. Chi-square test or Yates corrected chi-square test (when appropriate) was used to compare the accuracy of the three methods. The multivariate model was fitted using a generalized estimation equation (GEE) to estimate the accuracy of the three methods for cup and stem, respectively, adjusting for sex, surgical side, hip dysplasia, age, and BMI. The distribution of dependent variables was binomial with log as the link function. The templating time comparison among the three methods was performed using the GEE, and the dependent variable distribution was a Gaussian distribution. The significant level alpha was set to 0.05, and all statistical analyses were performed using R 3.6.3 software.

## Results

From October 2019 to July 2020, a total of 53 patients (29 men and 24 women) were included in the study. The mean age of the patients was 57.4 ± 1.7 years (27–79 years). There were 25 left and 34 right hips (59 hips). According to CHINA criteria, 6 (10%) hips were underweight, 33 (56%) were in the normal range, 13 (22%) were pre-overweight/obese, and 7 (12%) were obese. The primary diagnosis was DDH in 16 (27.12 %) hips, osteoarthritis (OA) in 16 (27.12%) hips, osteonecrosis of the femoral head (ON) in 16 (27.12%) hips, ankylosing spondylitis (AS) in 9 (15.25%) hips, and rheumatoid arthritis (RA) in 2 (3.39%) hips. OA, ON, AS, and RA were classified as non-DDH groups, 43 (72.88%) hips. The PINACLE cup (DePuy, Warsaw, IN, USA) was used in all hips, SUMMIT (DePuy, Warsaw, IN, USA) stem was used in 43 hips, and a CORAIL (DePuy, Warsaw, IN, USA) stem was used in 16 hips.

### Accuracy of prosthesis size

**Fig. 8:**
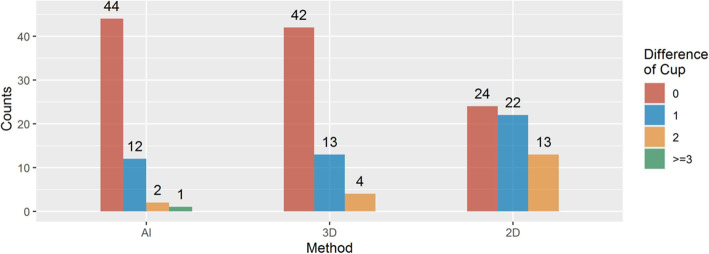
Prediction accuracy of cup of AI, 3D, and 2D methods

**Fig. 9:**
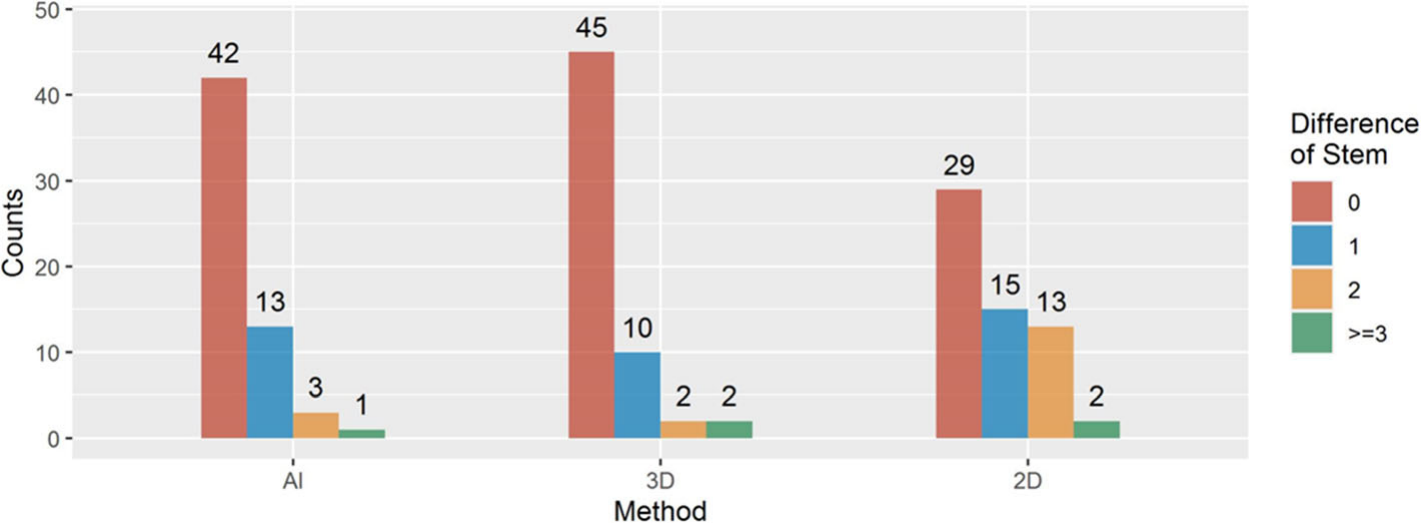
Prediction accuracy of stem of AI, 3D, and 2D methods

The accuracy of predicting size of acetabular cup and femoral stem components were 74.58% (44 of 59) and 71.19% (42 of 59), respectively, for AI HIP group; 71.19% (42 of 59) and 76.27% (45 of 59), respectively, for 3D mimics group; and 40.68% (24 of 59) and 49.15% (29 of 59), respectively, for 2D digital group (Figs. 8 and 9). The accuracy of AI HIP was significantly higher than that of the 2D digital template, with a statistical difference between the two methods (OR: 0.196 (0.087–0.441), *P* < 0.001 for cup; 0.377 (0.164–0.864), *P* = 0.021 for stem). Compared with the 3D mimics, the accuracy of AI HIP was comparable to that of the 3D mimics, with no significant difference between the two methods (cup: *P* = 0.743, stem: *P* = 0.468) (Tables 1 and 2).

**Table 1:** Generalized estimation equation model for predicting cup consistency rate of AI, 3D, and 2D methods

Factors	Coefficient	SE	Wald	*P* value	OR (95%CI)
(Intercept)	0.838	1.162	0.520	0.471	
AI	Ref				
3D	− 0.144	0.440	0.108	0.743	0.865 (0.365–2.051)
2D	− 1.63	0.414	15.504	< 0.001	0.196 (0.087–0.441)
Female	Ref				
Male	− 1.275	0.456	7.831	0.005	0.279 (0.114–0.682)
Left	Ref				
Right	0.166	0.392	0.179	0.672	1.181 (0.547–2.547)
DDH	Ref				
Non-DDH	1.550	0.499	9.637	0.002	4.71 (1.771–12.529)
Age (years)	− 0.003	0.013	0.047	0.828	0.997 (0.973–1.023)
BMI (< 18.5)	Ref				
BMI [18.5–23.9]	0.260	0.683	0.145	0.704	1.297 (0.34–4.95)
BMI [18.5–23.9]	− 0.222	0.737	0.090	0.764	0.801 (0.189–3.4)
BMI (≥ 28)	− 0.653	0.805	0.657	0.418	0.521 (0.107–2.523)

**Table 2:** Generalized estimation equation model for predicting stem consistency rate of AI, 3D, and 2D methods

Factors	Coefficient	SE	Wald	P value	OR(95%CI)
(Intercept)	− 0.709	1.141	0.385	0.535	
AI	Ref				
3D	0.331	0.456	0.526	0.468	1.392 (0.570–3.401)
2D	− 0.976	0.423	5.311	0.021	0.377 (0.164–0.864)
Female	Ref				
Male	− 0.759	0.397	3.643	0.056	0.468 (0.215–1.021)
Left	Ref				
Right	0.361	0.372	0.944	0.331	1.435 (0.693–2.971)
DDH	Ref				
Non-DDH	0.599	0.461	1.692	0.193	1.821 (0.738–4.492)
Age (years)	0.032	0.014	5.436	0.020	1.033 (1.005–1.061)
BMI (< 18.5)	Ref				
BMI [18.5–23.9]	− 0.312	0.650	0.230	0.632	0.732 (0.205–2.620)
BMI [18.5–23.9]	− 0.898	0.678	1.756	0.185	0.407 (0.108–1.538)
BMI (≥ 28)	0.565	0.850	0.442	0.506	1.76 (0.333–9.315)

### Time efficiency

The preoperative planned templating time of the AI group was 3.91 ± 0.64 min, 32.07 ± 2.41 min for the 3D group, and 2.96 ± 0.48 min for the 2D group. The templating time of AI HIP was 28.105 min shorter than that of the 3D mimics (*P* < 0.001), and 0.891 min longer than that of the 2D digital template (*P* < 0.001) (Table 3).

**Table 3:** Generalized estimation equation model for Time efficiency of AI, 3D, and 2D methods

Factors	Coefficient	SE	Wald	*P* value
(Intercept)	3.604	0.881	16.73	< 0.001
AI	Ref			
3D	28.105	0.279	10,158.119	< 0.001
2D	− 0.891	0.113	62.435	< 0.001
Female	Ref			
Male	0.470	0.197	5.712	0.017
Left	Ref			
Right	0.619	0.780	0.629	0.428
DDH	Ref			
Non-DDH	0.020	0.324	0.004	0.950
Age (years)	− 0.007	0.008	0.913	0.339
BMI (< 18.5)	Ref			
BMI [18.5–23.9]	− 0.044	0.394	0.013	0.910
BMI [24–27.9]	0.014	0.395	0.001	0.971
BMI (≥ 28)	0.168	0.531	0.101	0.751

### Factors influencing the accuracy of the AI HIP

#### Sex

The accuracy of acetabular cup in female patients was 76.00% (19 of 25) and male patients was 73.53% (25 of 34); there was no statistically significant difference between the two groups (*P* = 0.829). The accuracy of the femur stem in female patients was 68.00% (17 of 25) and male patients was 73.53% (25 of 34), and there was no statistically significant difference between the two groups (*P* = 0.643). It can be seen that sex has no effect on the accuracy of planning acetabular cup and femoral stem by AI HIP.

#### BMI

The accuracy of acetabular cup are as follows: normal group (18.5~23.9) was 78.79% (26 of 33), the overweight/obese group (24–27.9) was 69.63% (9 of 13), obese group (BMI ≥ 28) was 69.63% (4 of 7), and there was no statistically significant difference among the three groups (*P* = 0.600). Concerning the accuracy of the femur stem, the normal group (18.5~23.9) was 66.67% (22 of 33); the overweight/obese group (24–27.9) was 76.92% (10 of 13), and the obese group (BMI ≥ 28) was 85.71% (6 of 7). There was no statistically significant difference between the three groups (*P* = 0.725). Therefore, BMI has no effect on the accuracy of planning acetabular cup and femoral stem using AI HIP.

#### Hip dysplasia

Concerning the accuracy of acetabular cup, the DDH group was 50% (8 of 16), while the non-DDH group was 83.72% (36 of 43). The difference between the two groups was statistically significant (*P* = 0.021). As for the accuracy of the femoral stem, the DDH group was 50% (8 of 16), while the non-DDH group was 79.07% (34 of 43), respectively. There was no statistically significant difference between the two groups (*P* = 0.062). Therefore, in DDH patients, the accuracy of AI HIP to predict the acetabular cup seemed low, though, it had little impact on the accuracy of the femoral stem (Tables 4 and 5).

**Table 4:** Chi square test for the accuracy of predicting cup of AI method

Factors	Subgroup	*N*	Inaccurate (*n* = 15)	Accuracy (*n* = 44)	Chi-square	*P* value
Diagnosis (%)	DDH	16	8 (50.00%)	8 (50.00%)	5.328	0.021
	Non-DDH	43	7 (16.28%)	36 (83.72%)		
Sex (%)	Female	25	6 (24.00%)	19 (76.00%)	0.046	0.829
	Male	34	9 (26.47%)	25 (73.53%)		
BMI (%)	BMI (< 18.5)	6	1 (16.67%)	5 (83.33%)	1.869	0.600
	BMI [18.5–23.9]	33	7 (21.21%)	26 (78.79%)		
	BMI [24–27.9]	13	4 (30.77%)	9 (69.23%)		
	BMI (≥ 28)	7	3 (42.86%)	4 (57.14%)

**Table 5:** Chi square test for the accuracy of predicting stem of AI method

Factors	Subgroup	*N*	Inaccurate (*n* = 17)	Accuracy (*n* = 42)	Chi-square	*P* value
Diagnosis (%)	DDH	16	8 (50.00%)	8 (50.00%)	3.492	0.062
	Non-DDH	43	9 (20.93%)	34 (79.07%)		
Sex (%)	Female	25	8 (32.00%)	17 (68.00%)	0.215	0.643
	Male	34	9 (26.47%)	25 (73.53%)		
BMI (%)	BMI (< 18.5)	6	2 (33.33%)	4 (66.67%)	1.317	0.725
	BMI [18.5–23.9]	33	11 (33.33%)	22 (66.67%)		
	BMI [24–27.9]	13	3 (23.08%)	10 (76.92%)		
	BMI (≥ 28)	7	1 (14.29%)	6 (85.71%)		

## Discussion

Preoperative planning for THA has evolved from the widely used 2D digital template to 3D software planning, with a gradual improvement in the accuracy of prediction. However, most of the software operation procedures of 3D planning are still complicated and time-consuming at present. In this study, the AI HIP was used for preoperative planning of patients undergoing primary THA. The accuracy of acetabular cup and femoral stem prediction was 74.58% (44 of 59) and 71.19% (42 of 59), respectively, and the average templating time was 3.91 ± 0.64 min. Compared with the 3D mimics, the accuracy was similar, but the templating time was greatly shortened, making the operation more convenient. Compared with the traditional 2D digital template, its accuracy was much higher, with a slightly longer templating time than that of the digital template, but it can provide a 3D perspective that cannot be provided by the 2D digital template. In this study, AI technology was used for preoperative planning of THA for the first time and was compared with the accuracy and time efficiency of 3D and 2D planning. Combined with the current era of big data, the clinical application value of this technology in preoperative planning was established.

In order to highlight the objectivity of AI HIP, 3D mimics and 2D digital templates were planned and compared. In previous studies on 3D planning, Inoue et al. [17] conducted a retrospective study on 65 hips of 57 patients with a total of 65 hips using 3D planning software-zed-hip. The results showed that the complete accuracy of the femoral stem was 65%, 98% in one size, and 92% in the acetabular cup, and 100% in one size. Similarly, using the 3D planning software-zed-hip, Schiffner et al. [21] conducted a retrospective preoperative planning study on 116 patients. The results showed that the complete accuracy of the femoral stem was 58.6%, 94% in one size, and 56.9% in the acetabular cup and 86.2% in one size. The accuracy rate of 3D planning in the preoperative planning of complex hip joint diseases is also high. Zeng et al. [15] 3D mimics was used to study the preoperative planning of 20 cases of DDH patients with high dislocation. The results showed that the complete accuracy rate of acetabular cup was 70%, and that of one size was 100%, while the results of the traditional 2D template showed that the complete accuracy rate of the acetabular cup was 25%, and that of one size was 45%. Similarly, Peihui et al. [22] also used mimics to plan the acetabular cup in 41 patients with 49 hip DDH. The results showed that the complete accuracy was 71%, and 100% within a size. It can be seen that the above 3D preoperative planning-related research results show a high accuracy rate and has a great advantage in the planning of complex hip diseases. This is because 3D planning software provides different 3D perspectives. In our study, the results of 3D mimics planning showed that the complete accuracy of acetabular cup was 71.19%, and that of one size was 93.22%. The complete accuracy of the femoral stem was 76.27%, and that of one size was 93.22%, which was similar to the previous 3D planning results. The accuracy of the AI HIP also reached the prediction level of this 3D software (cup: complete accuracy was 74.58%, in one size was 94.92%; stem complete accuracy was 71.19%, in one size 93.22%). In previous studies on 2D digital templates, the accuracy of prosthesis prediction was low due to the influence of many factors. Strøm et al .[23] used a double-blind method to perform preoperative planning for 41 patients. The results showed that the complete accuracy rate of acetabular cup was 7.3%, that of one size was 41%, that of femoral stem was 34%, and that of one size was 76%. Shaarani et al. [24] carried out preoperative planning for 100 patients with the same digital template. The results showed that the complete accuracy rate of the acetabular cup was 38%, one size was 80%, and the complete accuracy rate of the femoral stem was 36%. Since the adjacent model of the accolade stem was 0.5, the internal size of one was 75%. Schiffner et al. [21] compared 3D and 2D planning methods, and the results showed that the complete accuracy rate of 2D planning acetabular cup was 44.8%, 45.7% for femoral stem, 56.9% for 3D planning of acetabular cup, and 58.6% for femoral stem. The above studies on 3D and 2D showed that the accuracy of 3D planning was significantly higher than that of 2D planning. In our 2D digital template planning, the results showed that the complete accuracy of the acetabular cup was 42%, including one size was 75%, the femoral stem was 50%, and including one size was 77%. The results of this study were similar to those of previous relevant literature. All plannings were checked by two experienced surgeons. If there was a disagreement, the planning was repeated by all the three surveyors agreed upon. Therefore, our results are both effective and reliable.

The AI HIP was found in 10 consecutive planned hip joints that the prediction of acetabular cup and femoral stem fluctuated in one size. In the first 10 patients planning, the accuracy of the acetabular cup and femoral stem was 70%. In the planning of 10–20 patients, the accuracy rates of acetabular cup and femoral stem were 80% and 70%, respectively. It shows that AI HIP was relatively stable, and there was no obvious deviation in the automatic learning in the early stage, so the planning could continue without elimination. In the whole study, there were 11 hips in one size, 2 hips in two sizes, and 1 hip in more than three sizes. In two hips with a difference of 2 sizes, one was osteoarthritis due to malunion of a chronic femoral neck fracture. Because of the shallow acetabular fossa and partial bone defect of the upper edge, the bone coverage of 54 mm cup was 87%, while the coverage reduced to 78% when using a 50-mm cup according to AI HIP planning. So AI HIP chose 54 mm as the optimal cup size in order to obtain a better bone coverage. However, when the acetabular cup was ground to 50 mm during the operation, it was found that the coverage rate of the acetabular cup was sufficient and the stability of the cup was satisfactory. Therefore, the grinding was not deepened, and a 50-mm cup was selected for implantation actually. In another patient, a 50-mm cup was implanted during the operation rather than a 54-mm cup which was an optimal size according to AI HIP planning. The possible reason was that the patient with ankylosing spondylitis because of ankylosis of the hip joint, the acetabulum was fused with the femoral head, and the software could not accurately segment the acetabulum and femoral head, resulting in a large deviation. Thus, the application of AI HIP in preoperative planning for serious ankylosing spondylitis needs further investigation. There was one case with a difference of more than 3 sizes. This patient had severe hip osteoarthritis with osteophytes around the hip joint. However, the software matched a 52-mm acetabular cup according to the true anterior and posterior diameter of the acetabulum, including the upper and lower diameter of the acetabulum. Perhaps the surrounding osteophytes interfered with the software in recognizing the acetabulum morphology. This led to a large error in the planning results. In the planning of the femoral stem, there were 10 hips in one size, 3 hips in two sizes, and 1 hip in three sizes. In 3 hips with a difference of 2 sizes, 2 hips were DDH, which may be due to: the variation of femoral neck anteversion angle and the increase of neck stem angle, the enlargement and upward movement of the femoral anterior arch, the enlargement of the anteroposterior diameter of the proximal femoral medullary cavity, and the reduction of transverse diameter. This may lead to the deviation of AI in identifying the epiphysis of the femoral shaft and the medial and lateral cortex of the distal femur, resulting in large planning errors, which requires AI HIP to make sufficient planning after a large number of DDH cases. It can determine the femoral variation of DDH, so that it can be more accurate in DDH. In the case of 3 sizes, the anterior femoral arch of the patient was significantly enlarged, which may have led to a bias towards matching the smaller femur stem during AI planning.

Our study also compared the templating time of the three planning methods. The results showed that the templating time of AI HIP was shorter than that of 3D mimics. The study of preoperative planning of the templating time has also been reported before, Schiffner et al. [21] compared the templating time of 3D and 2D for the first time. The results showed that the average templating time of the 2D template was 12 min (range 8–23 min), while that of the 3D template was 17 min (range 10–25 min). The templating time of 2D was faster than that of 3D, with an average of 5 min interval. When Sariali et al. [25] used hip-plan 3D planning software, the templating time was 10–15 min. Similarly, Inoue et al. [17] used a zed hip for preoperative planning, and the templating time was also 10–15 min. The reason for which their 3D templating time was faster than our 3D mimics templating time was because the algorithms of the pelvic plane, femoral morphology, and anatomical markers in their 3D software were automatic, while our 3D mimics was manual in pelvic segmentation, modeling, prosthesis implantation, and other operations. However, the AI HIP in our study was 3–5 times faster than the 3D software planning reported before, and 10 times faster than 3D mimics. This is because the software uses 3D segmentation neural network and 3D anatomical recognition neural network technology, which can quickly identify, segment, correct, and measurement by artificial intelligence, greatly shortens the templating time. Petretta [12] compared the time efficiency of the acetate template and digital template, and the results obtained showed that the templating time of the digital template was 154 s (range 73–343 s). In our study, the templating time of the 2D digital template was 2.96 ± 0.48 min, which was similar to the results of Petretta.

In the analysis of factors influencing the accuracy of AI HIP, we found that sex had no influence on the software. Holzer et al. [13] studied the accuracy of digital template in cementless prosthesis in 2D preoperative planning. The results obtained also showed that sex had no influence on the accuracy of digital template prediction because of little variation and limited differences in sex in the amplification rate of markers [26]. Previous studies on the influencing factors of BMI on the accuracy of 3D planning also found that obese patients were not affected by the accuracy of CT navigation to locate the acetabular cup [27]. In our study, AI HIP adds AI technology based on the CT data of patients. It is also based on the CT data for 3D planning, which is not affected by the magnification. Therefore, the accuracy of its prediction is not affected by BMI. In the analysis of hip dysplasia, we found that the accuracy of AI HIP in the DDH group was lower than that in the non-DDH group, and the difference was statistically significant. However, there was no significant difference in the accuracy between the DDH and non-DDH groups in the planning of the femoral stem. The main reason for these results was that 16 hips in the DDH group were Crowe I-II type, and the degree of femoral deformity variation was small. So, there was no significant difference in accuracy between the DDH and non-DDH groups. On the acetabular side, because in situ reconstruction of the cup, rather than appropriate upward or inward movement, is the first choice during AI HIP planning. While in the actual operation, 13 (81.25%) of the 16 hips in the DDH group had the acetabular cup appropriately moved upward or inward in order to achieve greater bone coverage. This may be the reason why the software was inaccurate in predicting acetabular cups in patients with DDH. Therefore, the software needs to expand the planning of the number of cases of each type of DDH, continuously strengthen the deep learning of the angle and position of the placement of each type of DDH acetabular cup, so as to master the placement rules of each type of DDH prosthesis and achieve accurate planning in DDH planning.

### Innovation

Based on AI technology and big data, compared with previous 2D and 3D studies, AI HIP has the following innovations: (1) speed: one-button intelligent operation, simple operation; (2) visualization: 3D visualized image output is immersive, providing realistic 3D visual anatomy; (3) pelvic correction: automatically corrects the position of the pelvis and lower limbs, without constant manual adjustment; (4) automatic planning: artificial intelligence planning can avoid planning deviation caused by personal experience, while the results of other 2D template methods and 3D template measurements are related to the experience level of the surveyors, and the more skilled the surveyors, the more accurate the results; (5) output scheme: generate a multi-perspective observation planning scheme, which is easy to realize in operation; (6) continuing education; further support and improvement of continuing education to shorten the doctor’s learning curve. At the same time, in this study, in order to avoid surgeon selection deviation, the blind method was adopted for the surgeon because if the surgeon participated in preoperative planning, the surgeon might change the grinding technique and insert a larger or smaller part by removing more or less bones when necessary, which will lead to errors.

### Limitations

(1) The prosthesis used in this study was limited to depuy products, and the range of prosthesis selection was small. (2) The sample size of the study was small, and the influencing factors of diseases were only divided into DDH and non-DDH groups, without further subdividing the difference in accuracy of the software in different diseases. (3) Only the accuracy of prosthesis size was evaluated, but important parameters such as rotation center, offset, and leg length discrepancy were not further evaluated. (4) Because of the need for CT scanning, the X-ray radiation exposure of patients was large, and the economic cost was also high. (5) CT data need to be copied, whether the data transmission process can be optimized or needs no further research. However, this is not the scope of this experiment. Next, we will continue to expand the cases, further study the accuracy of AI HIP in different diseases, evaluate its reproducibility in biomechanical planning and its impact on postoperative clinical efficacy.

## Conclusions

AI HIP showed excellent reliability for component size in THA. Acetabular dysplasia may affect the accuracy of AI HIP templating. Therefore, it is necessary to continue to expand the number of DDH cases and continuously strengthen the AI learning process.

## Data Availability

The datasets during and/or analyzed during the current study available from the corresponding author on reasonable request.

## Abbreviations

AI: Artificial intelligence
THA: Total hip arthroplasty
2D: Two-dimensional
3D: Three-dimensional
BMI: Body mass index
DDH: Developmental dysplasia of the hip
OA: Osteoarthritis
ON: Osteonecrosis
AS: Ankylosing spondylitis
RA: Rheumatoid arthritis
